# Preoperative transcatheter arterial embolization enables safe resection of a giant hypervascular pancreatic acinar cell carcinoma: A case report

**DOI:** 10.1016/j.radcr.2026.02.013

**Published:** 2026-03-14

**Authors:** Seiko Miura, Koji Nobata, Yoshisuke Kadoya, Tetsuya Minami, Yuka Nishino, Satoshi Shibata, Tamaki Kondo, Kiyotaka Ohta, Takafumi Mochizuki, Sohsuke Yamada, Nobuhiko Ueda

**Affiliations:** a The Department of Radiology, Kanazawa Medical University, Ishikawa, Japan; b The Department of Pathology, Kanazawa Medical University, Ishikawa, Japan; c The Department of General and Digestive Surgery, Kanazawa Medical University, Ishikawa, Japan; d The Department of Infectious Diseases, Kanazawa Medical University, Ishikawa, Japan

**Keywords:** Pancreatic acinar cell carcinoma (ACC), Preoperative Transcatheter Arterial Embolization (TAE), Hypervascular Pancreatic Tumors

## Abstract

Pancreatic acinar cell carcinoma (ACC) is a rare malignant neoplasm that accounts for 0.4%-0.7% of all pancreatic tumors. It often presents as a large, bulky mass owing to its expansive growth pattern. We report a case of a large pancreatic ACC that achieved remarkable long-term recurrence-free survival after successful surgical resection supported by preoperative interventional radiology (IR).

A 64-year-old male presented to our hospital with weight loss and abdominal distension. A firm mass was palpable in the left upper abdomen. CT revealed a giant, heterogeneously enhancing tumor measuring 16.8 cm. Because of the anticipated massive intraoperative hemorrhage associated with tumor size and hypervascularity, Transcatheter Arterial Embolization (TAE) was performed preoperatively. The bilateral inferior diaphragmatic artery, posterior gastric, and splenic arterial branches supplying the tumor were embolized using metal coils and embolic materials.

A safe radical resection was successfully performed (distal pancreatectomy, splenectomy, partial gastrectomy, partial colon resection, and left adrenalectomy). The pathological diagnosis confirmed pancreatic ACC (stage IIB). The patient has maintained recurrence-free survival for more than 5 years postoperatively.

This case highlights that aggressive surgical resection achieves long-term survival in large pancreatic ACCs. Preoperative IR-TAE effectively controlled bleeding risk, underscoring the crucial role of this technique in safely managing high-risk, large, hypervascular pancreatic tumors. Physicians must consider ACC as a differential diagnosis for large pancreatic masses that may mimic other cystic solid lesions, such as Intraductal Papillary Mucinous Neoplasm (IPMN). Close multidisciplinary collaboration, particularly between interventional radiology and surgery, is essential in managing these challenging cases.

## Introduction

Pancreatic acinar cell carcinoma (ACC) is a rare epithelial malignancy of the exocrine pancreas, accounting for 0.4%-0.7% of all pancreatic neoplasms [1]. ACC frequently presents as a large tumor at diagnosis, often measuring more than 10 cm [2]. Although the prognosis of resectable ACC is generally more favorable than that of pancreatic ductal adenocarcinoma (PDAC), curative treatment relies on radical surgical resection with negative margins [3].

Recent comprehensive reviews highlight that ACC is biologically distinct from PDAC and often exhibits hypervascularity, expansive growth, and large tumor volume at presentation [4].

Histopathological studies further demonstrate rich capillary networks within ACC, correlating with its hypervascular imaging appearance [5]. Typical radiologic features include a well-circumscribed, expansile mass with heterogeneous enhancement, which may complicate differentiation from other solid pancreatic tumors [6].

Managing giant ACCs is particularly challenging due to their size, vascularity, and proximity to major visceral structures, all of which increase the risk of massive intraoperative hemorrhage. Although preoperative transcatheter arterial embolization (TAE) is not routinely used for ACC, it may mitigate bleeding risk in selected hypervascular tumors.

We describe a rare case of a giant hypervascular ACC safely resected following preoperative TAE, resulting in long-term recurrence-free survival. This case underscores the importance of coordinated interventional radiology and surgical planning when treating large, hypervascular pancreatic tumors.

## Case presentation

A 64-year-old male presented with weight loss and abdominal distension with a 10 kg weight reduction over 1 year. Physical examination revealed a large, firm mass in the left upper abdomen, measuring ≧ 10 × 10 cm.

Admission blood tests: Complete blood count showed Hb 14.0 g/dL with no anemia; biochemical tests showed CRP 0.04 mg/dL with no signs of inflammation. The tumor markers levels were CEA 6.4 ng/mL, CA19-9 81.6 U/mL, AFP 42.9 ng/mL, DUPAN-2 43 U/mL, Span-1 24 U/mL, all showing mild elevation.

**Fig. 1:**
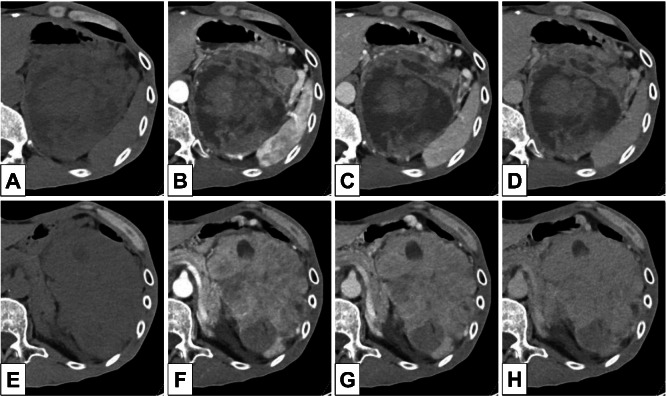
Contrast enhanced dynamic CT images. A well-defined tumor is present in the left upper abdomen. (A) Non-contrast CT of the upper portion of the tumor. (B) Arterial phase of the upper portion of the tumor. (C) Portal venous phase of the upper portion of the tumor. (D) Venous phase of the upper portion of the tumor. (E) Non-contrast CT of the lower portion of the tumor. (F) Arterial phase of the lower portion of the tumor. (G) Portal venous phase of the lower portion of the tumor. (H) Venous phase of the lower portion of the tumor. The upper portion of the tumor predominantly contains areas of fluid density, which do not enhance. The lower portion predominantly contains soft tissue density, which enhances in the arterial phase and shows diminished enhancement in the venous phase.

Computed tomography (CT) Findings: Axial CT showed a mass with a maximum diameter of 16.8 cm in the left upper abdomen, which was suspected to originate from the pancreas. The tumor displayed heterogeneous density, including low-density areas, suggesting necrosis or cystic degeneration in the superior portion, and heterogeneous solid density in the caudal portion. The tumor was in close contact with the gastric wall and displaced the pancreatic body/tail and left kidney, with partially indistinct borders at the interface between the stomach and pancreatic tail (Fig. 1). In the coronal views the cephalic portion of the tumor displaced the left diaphragm upward, while its caudal portion displaced the left kidney and renal vessels caudally.

Dynamic contrast-enhanced CT confirmed a hypervascular nature. Most tumors, except for the low-density area, demonstrated strong enhancement from the early phase, albeit with heterogeneous enhancement, suggesting a highly vascular tumor. The tumor compressed the stomach, pancreatic body/tail, and left kidney. Components with strong enhancement were comparable to those of the pancreatic parenchyma. The tumor was located in the pancreatic tail and in contact with it, although not extensively. The tumor infiltration did not extend to the splenic artery bifurcation, and the spleen was displaced dorsolaterally. The tumor was also in contact with the small intestine and descending colon; however, no obstruction was observed.

**Fig. 2:**
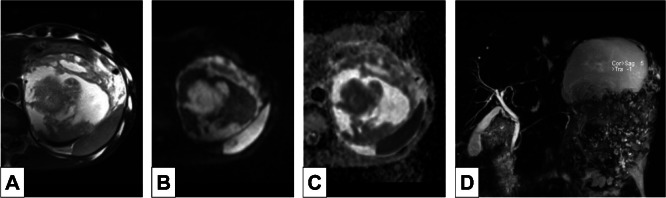
MRI. (A) T2-weighted image (T2WI). (B) Diffusion-weighted image (DWI) (b = 800). (C) Apparent diffusion coefficient (ADC) map. (D) Magnetic resonance cholangiopancreatography (MRCP). The hyper intense portions of the tumor on T2WI show no diffusion restriction and also appear hyper intense on MRCP. The hypointense portions of the tumor on T2WI show diffusion restriction. No dilatation of the main pancreatic duct is seen on MRCP.

MRI: The solid component shows heterogeneous low-signal intensity on T1-weighted images, heterogeneous mildly high-signal intensity on T2-weighted images and marked diffusion restrictions on diffusion-weighted images (Fig. 2A). Dynamic MRI showed early enhancement of the tumor, similar to dynamic CT, with enhancement appearing more heterogeneous.

**Fig. 3:**
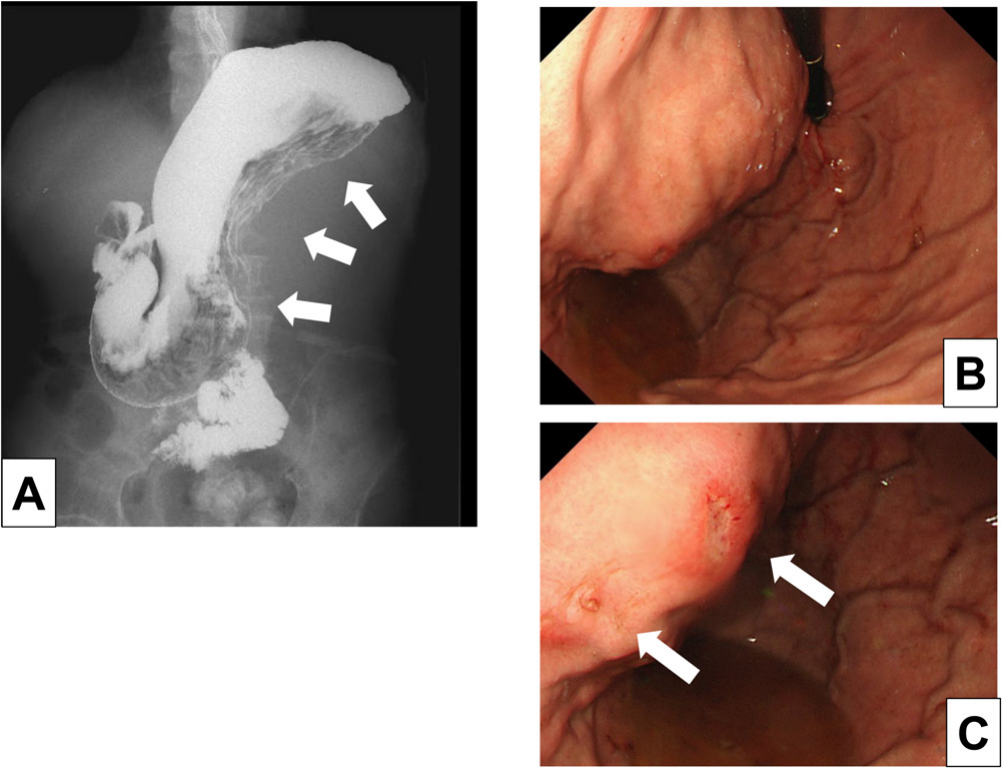
Upper GI fluoroscopic image using Gastrografin. (A) The body to the fundus of the stomach was displaced posteriorly from the greater curvature due to the mass. Upper GI Endoscopic Image. (B) The posterior wall of the upper gastric body showed an SMT-like-or extramural compression-like elevation. (C) A 5 mm erosion was present on the surface of the elevation; however, no malignant findings were identified on biopsy.

Upper GI fluoroscopic image findings using Gastrografin: The body to the fundus of the stomach was displaced posteriorly from the greater curvature due to the mass (Fig. 3A).

Upper GI endoscopic findings: The posterior wall of the upper gastric body showed an SMT-like-or extramural compression-like elevation (Fig. 3B). Additionally, a 5 mm erosion was present on the surface of the elevation; however, no malignant findings were identified on biopsy (Fig. 3C).

**Fig. 4:**
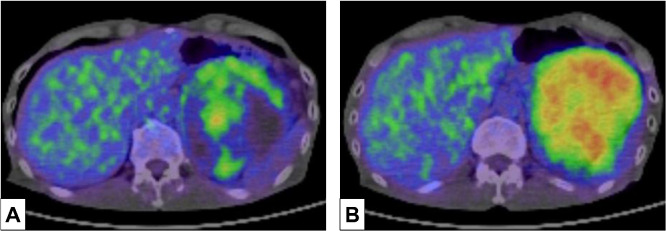
FDG PET/CT images. (A) The upper portion of the tumor. FDG uptake is observed in the soft tissue density areas of the tumor and is sparse in the fluid density areas. (B) The lower portion of the tumor. High FDG uptake is observed in the tumor.

¹⁸F–FDG-PET/CT Findings: The solid tumor component showed accumulation of fluorodeoxyglucose F18 (18F-FDG) with early (SUV max) of Early = 9.61 and Delay (14.35) maximum standardized uptake values. No uptake was observed in the other areas (Fig. 4). 18F-FDG uptake was noted at the tumor margin, with a maximum standardized uptake value (SUV max) of 11.96 (Figs. 4A and B).

Owing to the imaging features, the origin of the tumor was initially ambiguous. Differential diagnoses included pancreatic masses (eg, ACC and pancreatic neuroendocrine tumor [pNET]),

Gastrointestinal Stromal Tumor (GIST), and malignant lymphoma. While pNET shares a similar hypervascular enhancement pattern with ACC, the lobulated appearance and extensive internal necrosis observed in this case are atypical for pNET. Furthermore, the tumor’s massive size (16.8 cm) and the notable absence of main pancreatic duct dilatation strongly favored a diagnosis of ACC over other solid pancreatic neoplasms. These specific imaging characteristics were instrumental in prioritizing ACC within the differential diagnosis.

**Fig. 5:**
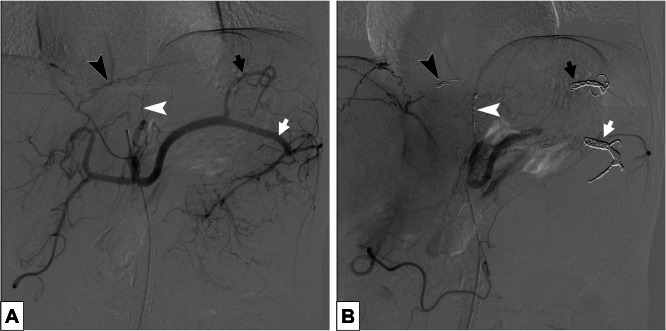
Angiography images. (A) Initial celiac artery angiography. (B) Post-embolization celiac artery angiography. The branches of the splenic artery (white arrow), the short gastric artery (black arrow), the left inferior phrenic artery (white arrowhead) and the right inferior phrenic artery (black arrowhead) were embolized.

Preoperative TAE was performed 1 day before surgery to reduce the risk of massive intraoperative hemorrhage due to the tumor’s extreme size and marked hypervascularity. Angiography confirmed the hypervascularity of the mass, with extensive tumor vessel networks and early tumor staining. The initial IR findings suggested a primary origin in the pancreatic tail because the splenic artery was identified as the main feeding vessel supplying the pancreatic tail; however, a definitive preoperative diagnosis was not reached. The main feeders, including the branches from the bilateral inferior diaphragmatic, posterior gastric, and splenic arteries, were successfully embolized using metal coils and embolic materials (Figs. 5A and B). The right inferior phrenic artery was embolized with 1 Target XL 360 Soft coil (3 mm × 9 cm), and the left inferior phrenic artery with 1 Target XL 360 Soft coil (2 mm × 6 cm). The short gastric artery and the caudal portion of the splenic artery were embolized using 2 Target XXL 360 coils (6 mm × 40 cm) in combination with Embosphere microspheres (100-300 μm). Subsequently, the splenic artery main trunk from distal to proximal segments, including partial side branches, was embolized with Target XXL 360 coil (8 mm × 40 cm). The embolization endpoint was confirmed by celiac angiography, which demonstrated a marked reduction in tumor blush. Feeder vessels originating from the jejunal branch were observed but were deemed too difficult to embolize. Angiography via the superior mesenteric artery revealed numerous reticular tumor feeding vessels arising from jejunal branches, embolization of these vessels was considered technically difficult and was therefore not attempted. No post-embolization syndrome or embolization-related complications were observed.

**Fig. 6:**
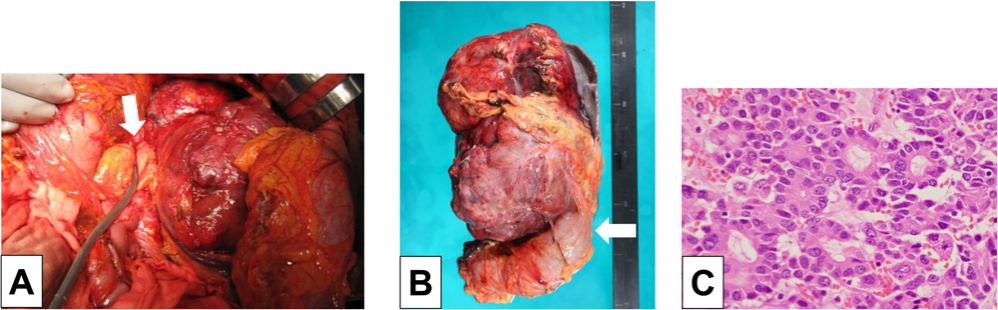
Specimen photographs (A) Intraoperative image. The resection was performed at the pancreatic body-tail junction (white arrow). (B) Resected specimen (white arrow: colon). (C) Histopathological image (haematoxylin–eosin stain).

During laparotomy, the tumor measured 15 cm × 10 cm and was excised en masse along with the adherent organs (Fig. 6A and B). Complex surgical procedures included distal pancreatectomy, splenectomy, partial gastrectomy, partial resection of the descending transverse colon, and left adrenalectomy. Preoperative TAE successfully suppressed intraoperative hemorrhage and no intraoperative blood transfusion required.

Histological examination revealed that the mass was confined with a clear boundary. Pathological findings revealed tumor cells forming acinar-like structures with eosinophilic granular cytoplasm and large nuclei, consistent with Acinar Cell Carcinoma (Fig. 6C). The final pathological diagnoses were ACC, pT3, pN1 (1/1 retrieved lymph nodes), M0, and pStage IIB.

The patient had received adjuvant chemotherapy (nab-paclitaxel plus gemcitabine) for 2 years. The patient has remained recurrence-free for 5 years postoperatively.

## Discussion

This case is remarkable for both the tumor’s large size (15 × 10 cm) and the patient’s long-term recurrence-free survival. ACC often presents as a large mass, with mean reported tumor sizes around 10 cm [2], and the present case lies at the upper end of this range. Complete resection is associated with improved survival [3], making safe operative planning essential. A key feature of ACC is its hypervascularity, resulting from dense intratumoral capillary networks [5].

This was evident in our case through intense arterial-phase enhancement on CT/MRI and numerous CD31-positive vessels on pathology. Hypervascularity in ACC has also been emphasized in broader clinical reviews [7]. Preoperative TAE was selected as a bleeding-risk reduction strategy because of the tumor’s extreme size and pronounced hypervascularity. The procedure was intentionally performed 1 day before surgery to achieve sufficient devascularization while minimizing the risk of prolonged ischemia or inflammatory changes prior to resection. Although the literature confirming TAE for hemorrhage prevention in pancreatic ACC is limited, the empirical application in this case proved effective in minimizing bleeding and ensuring surgical safety, successfully controlling TAE was well tolerated, with no post-embolization syndrome or procedure-related complications, and contributed to safe multivisceral resection under controlled operative conditions (intraoperative blood loss to 940 mL and an operation time of 4 hours and 55 minutes).

Radiologically, ACC typically appears as a large, well-circumscribed, heterogeneously enhancing mass with expansile growth [6], consistent with the present case. ACC exhibits a broad morphological spectrum—including cystic, solid, and mixed patterns—which may mimic other pancreatic tumors such as neuroendocrine tumors or solid pseudo papillary neoplasms [8]. Moreover, intraductal polypoid variants of ACC have been reported to resemble intraductal papillary mucinous neoplasms (IPMN), complicating diagnosis [9].

ACC also demonstrates molecular heterogeneity. BRCA2 germ-line mutations and NTRK gene fusions have been reported in a subset of cases, offering potential avenues for targeted therapy [10,11]. Although molecular analysis was not performed in our patient, these findings highlight the biological diversity of ACC.

This case underscores the importance of meticulous preoperative planning and close collaboration between interventional radiologists and surgeons. The combination of selective TAE and coordinated surgical strategy played a central role in achieving safe tumor resection and excellent long-term disease control.

## Conclusion

We report a rare case of a giant hypervascular ACC successfully treated by preoperative TAE followed by multivisceral resection, resulting in more than 5 years of recurrence-free survival. TAE may reduce bleeding risk and facilitate curative surgery in large hypervascular pancreatic tumors. ACC should be considered in the differential diagnosis of large pancreatic masses, including IPMN mimicking lesions.

Learning points

• Pancreatic acinar cell carcinoma (ACC) typically presents as a large, well-circumscribed mass with intense arterial-phase enhancement. However, its morphological diversity—including intraductal polypoid variants—can closely mimic other lesions such as IPMN, necessitating a broad differential diagnosis.
• Selective TAE effectively reduces arterial inflow and tumor blush, significantly mitigating bleeding risks and enabling the safe, curative resection of giant hypervascular tumors that might otherwise be considered high-risk for surgery.
• A meticulous radiological evaluation is crucial for identifying hypervascularity and extensive feeder vessel networks. This assessment allows for the identification of high-risk cases where massive intraoperative hemorrhage is anticipated.

Alt-text: Unlabelled box dummy alt text

## Ethical approval

This study was approved by the Ethics Committee of our institution.

## Patient consent

Informed consent was obtained for the case report to be published.
